# Osteogenesis Imperfecta with Celiac Disease and Type II Diabetes Mellitus Associated: Improvement with a Gluten-Free Diet

**DOI:** 10.1155/2012/813461

**Published:** 2012-03-05

**Authors:** Luis Rodrigo, Isabel Pérez-Martinez

**Affiliations:** Gastroenterology Service, Hospital Universitario Central de Asturias (HUCA), University of Oviedo, c/Celestino Villamil s. no. 33006, Oviedo, Spain

## Abstract

Osteogenesis imperfecta (OI) is a genetic disease, with a connective tissue alteration, consisting in the presence of multiple spontaneous fractures or after minimal traumatism. Its association with other metabolic processes is rarely described. We present the clinical case of a female adult patient of 43 years. From her infancy, she has had multiple fractures, needing several surgical interventions, and she was diagnosed of OI type 2 at adolescence age. Due mainly to difficulties in walking remaining in wheel-chair in the last three years, she was overweight with morbid obesity (BMI = 45.4) and had a type-II DM associated. She suffered from recurrent abdominal pain and chronic diarrhea and was diagnosed of celiac disease (CD) with increased intraepithelial duodenal infiltration, being classified as lymphocytic enteritis, Marsh I type. She was put on a gluten-free diet (GFD), having lost 6 kg of weight after 6 months, with a good control of DM-II and presenting a significant clinical improvement. It is rewarding to search the presence of two coincidental metabolic diseases associated to OI, specially CD, because of the dramatic clinical benefit in the general found after putting on a GFD.

## 1. Introduction

Osteogenesis imperfecta (OI) is a genetic disease, associated with a connective tissue disorder, related to different phenotypic presentations. It is also called “brittle bone disease.” Severely affected patients have multiple fractures since childhood, both spontaneous or related to minimal trauma, and the most seriously affected children usually die in the neonatal period. Milder forms of OI may be manifested later on, in the form of early osteoporosis or bone mineral loss, that is more important after the menopause [1, 2].

Its association with other metabolic processes is rarely described. We present here the case of an adult woman with a severe OI, diagnosed in the adolescence period and more recently of celiac disease (CD) and type II diabetes mellitus (DM-II), describing its evolution and changes after starting on a gluten-free diet (GFD).

## 2. Case Presentation

**Figure 1:**
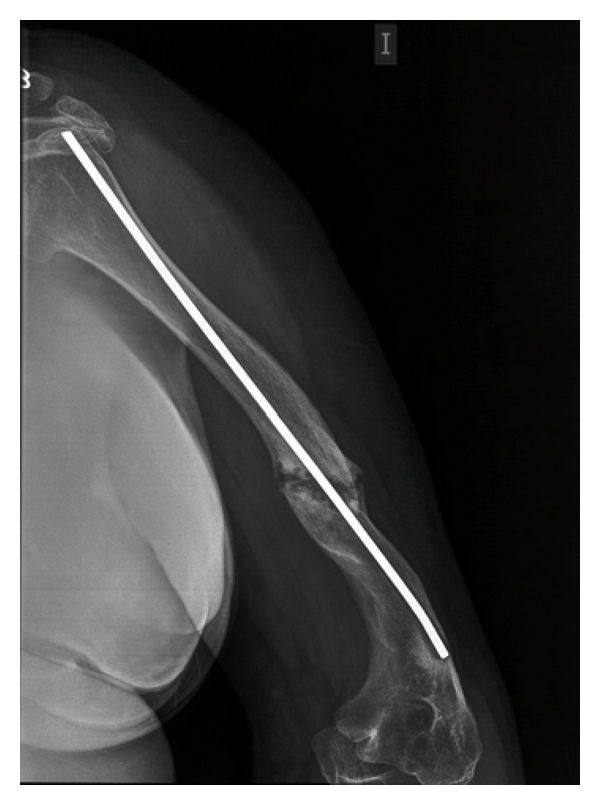
Intramedullary nail placed on the left humerus fracture.

We present the case of a 43-year-old female. Shortly after birth, she presented a spontaneous right hip fracture. From then, until now, she has had multiple fractures of both arms and legs, which have required several interventions for correction, needing on the left humerus to be fixed with a medullary locking (Figure 1). The last fracture episode happened 3 years ago. OI type 2 was diagnosed, 20 years ago. For moving at home, she walks with difficulty with a cane, and, on the street, she is moved by wheelchair. She presents a significant growth delay (High = 1′14 m) and weighed 65 kg, presenting a picture of morbid obesity, with an increased body mass index (BMI = 45.4 kg/m^2^).

She relates that for about 2 years, and she has slow digestion, accompanied by marked abdominal bloating, accompanied by heartburn and reflux. The major alteration in her bowel habit was an alternating pattern with diarrhea and constipation. The patient had also bilateral hearing loss, requiring hearing aids for ten years.

Currently, the CBC was normal. Blood glucose was elevated (230 mg/dL) with increased glucosuria (4+). The rest of biochemical analysis was normal. She underwent a gastroscopy that showed a moderate reflux oesophagitis (grade B Angeles classification) without associated hiatal hernia. Duodenal biopsies showed an increased intraepithelial lymphocytosis (45%) corresponding to stage I of affordable Marsh. Genetic markers of CD type DQ2 were positive in homozygosis, and antibodies to tissue transglutaminase (tTG) were normal (0.6 U/mL). Antinuclear antibodies were negative. Vitamin D deficiency was excluded.

## 3. Intervention

She was started on a gluten-free diet (GFD) and an oral antibiotic drug, type Metformine-850 mg/twice daily was given, having lost 6 kg of weight in 6 months with good control of DM-II and presenting a significantly improvement related to the presence of prior digestive problem. A recently completed study of bone mineral density (BMD) has been reported as normal.

## 4. Discussion

The estimated incidence of OI is approximately one case per live 20,000 births. This means that it is included within the so-called orphan diseases as defined in the USA when the disease affects fewer than 200,000 people. There are over 200 genetic mutations associated with the phenotype of OI [3].

Most OI patients have mutations that affect one or both chains of type I collagen, although up to 10% of patients do not have them. The defect in bone quality explains many of the clinical aspects of OI.

The most common clinical manifestations include the presence of multiple fractures, associated by short stature, scoliosis, deformities of the skull base, blue sclerae, hearing loss, opalescent teeth, increased laxity of ligaments and skin, and presence of spontaneous bruising. The frequency of fractures decreases after the puberty [4–6]. The diagnosis of OI is based on signs and symptoms listed above. A positive family history is a factor of great help. However, in the absence of these events, it can be difficult to achieve. Extraskeletal manifestations may be subclinical (deafness, blue sclerae, or defective dentinogenesis).

Although the biochemical parameters are normal, some alterations may be present, such as high levels of alkaline phosphatase; the presence of hypercalciuria is common in children, together with increased bone resorption markers [7, 8]. Their association in adults with CD and DM-II is poorly understood, but its diagnosis and treatment significantly improve the quality of life of patients and therefore to her clinical and/or analytical, it is advisable to perform an adequate search of these diseases, for their early identification and treatment [9, 10].

In conclusion, we must emphasize the convenience of ruling out the presence of a gluten intolerance in patients with osteogenesis imperfecta, mainly if they present a type II diabetes mellitus associated, and the clear clinical benefit observed on a GFD, as it has been shown in the presented case.
